# Efficient Data Communication Using Distributed Ledger Technology and IOTA-Enabled Internet of Things for a Future Machine-to-Machine Economy

**DOI:** 10.3390/s21134354

**Published:** 2021-06-25

**Authors:** Mohd Majid Akhtar, Danish Raza Rizvi, Mohd Abdul Ahad, Salil S. Kanhere, Mohammad Amjad, Giuseppe Coviello

**Affiliations:** 1 Department of Computer Engineering, Jamia Millia Islamia, New Delhi 110025, India; akhtarmajid273@gmail.com (M.M.A.); drizvi@jmi.ac.in (D.R.R.); mamjad@jmi.ac.in (M.A.); 2 Department of Computer Science and Engineering, Jamia Hamdard, New Delhi 110062, India; itsmeahad@gmail.com; 3 School of Computer Science and Engineering, University of New South Wales, Sydney, NSW 2052, Australia; salil.kanhere@unsw.edu.au; 4 Department of Electrical and Information Engineering, Polytechnic University of Bari, 70126 Bari, Italy

**Keywords:** IoT, blockchain, DLT, IOTA, communication, security, privacy, scalability

## Abstract

A potential rise in interest in the Internet of Things in the upcoming years is expected in the fields of healthcare, supply chain, logistics, industries, smart cities, smart homes, cyber physical systems, etc. This paper discloses the fusion of the Internet of Things (IoT) with the so-called “distributed ledger technology” (DLT). IoT sensors like temperature sensors, motion sensors, GPS or connected devices convey the activity of the environment. Sensor information acquired by such IoT devices are then stored in a blockchain. Data on a blockchain remains immutable however its scalability still remains a challenging issue and thus represents a hindrance for its mass adoption in the IoT. Here a communication system based on IOTA and DLT is discussed with a systematic architecture for IoT devices and a future machine-to-machine (M2M) economy. The data communication between IoT devices is analyzed using multiple use cases such as sending DHT-11 sensor data to the IOTA tangle. The value communication is analyzed using a novel “micro-payment enabled over the top” (MP-OTT) streaming platform that is based on the “pay-as-you-go” and “consumption based” models to showcase IOTA value transactions. In this paper, we propose an enhancement to the classical “masked authenticated message” (MAM) communication protocol and two architectures called dual signature masked authenticated message (DSMAM) and index-based address value transaction (IBAVT). Further, we provided an empirical analysis and discussion of the proposed techniques. The implemented solution provides better address management with secured sharing and communication of IoT data, complete access control over the ownership of data and high scalability in terms of number of transactions that can be handled.

## 1. Introduction

It is a fact that we live in a far more complex digital age than what we use to only a few decades ago. The digital era has acquired enormous importance around us as well as in our lives, ranging from handheld devices like mobile phones, tablets or laptops to home devices like Alexa, Google-mini, smart A.C., smart T.V., etc.

The Internet of Things is the concoction of communication within devices with other devices in the physical real world. All smart internet protocol (IP)-enabled devices such as cameras, thermostats, Fitbit devices, smart geysers, or smart A.C. work in a complimentary fashion sharing the real-world data between them. For example, when a person enters a home, sensors can identify his presence or a camera can recognize him and convey an instruction to the A.C. to turn on in accordance with that user’s preferred temperature [1]. IoT refers to the assimilation of devices when harnessed with the internet bubble [1,2]. According to reports from Statista (https://www.statista.com/statistics/1183457/iot-connected-devices-worldwide/, accessed on 22 January 2021) and IoT-Analytics (https://iot-analytics.com/state-of-the-iot-update-q1-q2-2018-number-of-iot-devices-now-7b, accessed on 20 December 2019), it is estimated that by 2025, there will be close to 22 billion devices connected to the internet, representing an annual increase of about 21% (https://www.gartner.com/en/newsroom/press-releases/2019-08-29-gartner-says-5-8-billion-enterprise-and-automotive-io, accessed on 5 November 2019).

The Internet of Things (IoT) is no longer a futuristic concept, instead it is already here. Many startups and companies are building solutions around the IoT. With the help of these innovations, we can now control the devices, appliances, and gadgets in our homes just from our smartphones from anywhere around the globe through the internet.

### 1.1. Problem Description

Current global supply chain and logistics are continuously facing multiple complications due to lack of transparency, trust and social sustainability involved in distributed businesses [3,4]. To mitigate such issues of trust and transparency in distributed networks, solutions like distribute ledger technology (DLT) (including blockchain in particular) combined with IoT can help the network to increase traceability and overall reliability. Also, in the present IoT implementation the data is highly unreliable and most of the time data integrity is not ensured [5,6]. In present-day critical applications like healthcare, military operations, weather forecasting, etc., data reliability is of prime importance since they work based on data-driven decision making. Thus, we need a reliable system to ensure data integrity. This paper aims to give the insights of future of distributed ledger technologies like IOTA applied on IoT.

Blockchain itself can be realized as either a public blockchain (Ethereum) or a private blockchain (Hyperledger Fabric). Blockchain and IoT are a perfect fit for each other. In the past, blockchain-based systems have proven to be highly secure systems. However, there are many factors which prevents the widespread adoption of blockchain-based systems like their low number of transactions per second (TPS), scalability issues and transaction fees [3]. In this paper, we presented our solution for eradicating these problems with other DLT solutions like IOTA [7].

### 1.2. Motivation and Problems with Current IoT Implementations

IoT applications come with lot of features like being omnipresent and pervasive. However, the IoT ecosystem is vulnerable due to a number of factors [5]. Major problems seen in IoT implementations are:

- *Highly centralized systems*: Being centralized might not be an issue in today’s internet world but being the single point of failure increases the probability of concerns. Also, it is no hidden fact that such centralized authoritarian systems often pose issues like data leakage, transparency, availability, etc. Further, there is a high chance of data being sold to third parties without the consent of the data owner [8].
- *Data privacy and access control*: IoT data or personal identifiable information (PII) is generally stored in raw format in the cloud storage or uses rudimentary encryption mechanisms which are easy to break. The existing system is always vulnerable to the trust and privacy issues. Hence, a reliable system is needed to secure the IoT implementation with respect to data privacy, access control and integrity [9].
- *Data Integrity and Authenticity*: The current implementations of IoT devices are hardcoded with pre-configured weak passwords. Such limited security designs provided by IoT manufacturers attract attackers/hackers the most. Moreover, these systems are not considered reliable due to the absence of digital signatures to prove the integrity of the data packets [5].
- *Identity and data management*: It is a key challenge to securely store data and to prevent it from any unauthorized access. With enormous amounts of devices associated with each person or thing, management of identities and data generated by these devices are often neglected in current IoT implementations. DLT systems have proven to be fit for storing information securely. With DLT, we can be sure of knowing if the settings have breached or tampered with [1].

The urgency for IoT security is increasing day by day. Even ahead of security, a system needs to be fast and reliable at all times. It must be highly scalable to handle the transmitted data for each second or microseconds from all the number of IoT device connected. Data reliability is the need of the hour. Multiple cases are seen where if IoT devices are compromised, they can lead to unprecedented situations including distributed denial of service (DDoS) attacks, spam attacks, etc. In the recent past, similar kind of DDoS particularly happened from Mirai Botnet in July 2016 on Dyn, a domain naming company which observed an uncontrollable speed of 665 Gigabytes per second (https://www.flashpoint-intel.com/cybercrime-forums-fraud/action-analysis-mirai-botnet-attacks-dyn/, accessed on 10 May 2019). After hours of logging and debugging, later it was found that the attack was programmed by the Mirai Botnet using just internet protocol-enabled security cameras, routers and printers at its disposal. Alternatively, integration of Internet-enabled devices such as mobile phones, Arduino Uno or Raspberry Pi with the DLT will lead a new way to the provide reliability, transparency and traceability of the DLT ecosystem. The advantage will not only be in the supply chain domain but also in many other areas like smart homes where electricity can be shared, or in the automotive industry for finding car parking spaces and directly paying through machine/car wallets, but the industry that will benefit the most will be pharmaceutical industry where the sensitive data such as temperature or humidity can be recorded on the DLT for complete trust between all the participants in the pharma supply chain, thus providing a more trustable ecosystem [3]. Taking the above stated issues as motivation, in this paper we have explored the use if the IOTA platform for pushing and fetching data from the IOTA tangle protocol as a solution for scalability.

### 1.3. Problem Definition

The current-information era is diverse and immersive where the data is being generated at an exponential rate. The IoT is one of the fastest growing industries in the technological space. With such high demand and deployment of IoT devices, it fails to prove its suitability for a number of parameters. These include quality of data, trust, reliability, ownership, security and privacy [10,11,12]. The built-in security in IoT devices for the communication with the internet is not based on cryptography. Generally IoT device implementations lack end-to-end encryption and decryption schemes. Even if some security schemes are deployed in the hardware, the overall system remains vulnerable to different types of attacks.

### 1.4. Our Contributions

This paper offers detailed research on IOTA convergence with the IoT. We have explored in depth the use of the IOTA platform for real-time IoT applications starting from the preliminaries, architecture, components and methodology for communication protocol used in our IoT case scenario. The major contributions of the paper are as follows:

- *It explores the role of Blockchain and DLT in the IoT ecosystem and the Machine-to-Machine (M2M) Economy.*

An exhaustive discussion on the convergence of blockchain (Ethereum, Hyperledger) and DLT (IOTA platform) has been done in this paper. The approach provides a realistic view with a focus on the real world adoptability and evaluation of these techniques (see Section 2).

- *It provides detailed insights into IOTA platform ecosystem Version 1 (before Coordicide) for IoT applications.*

The paper discusses exclusively the next generation blockchain i.e., IOTA platform ecosystem and dissects the components of the IOTA platform including the masked authenticated message (MAM) protocol which is used for sharing messages and data between IoT devices securely (see Section 3).

- *A novel Dual Signature Masked Authentication Message (DSMAM) is proposed by enhancing the classical IOTA ‘masked authentication message (MAM) version 0.x (v0) communication protocol’ protocol using a second digital signature scheme (Ed25519) layer.*

We propose to add the Edwards Curve Digital Signature scheme (Ed25519) for verifications over the shared masked authenticated message (MAM) communication protocol and implement secured and verified data channels using MAM and IOTA Tangle. The proposed DSMAM is providing similar performance to the classical IOTA MAM with additional enhanced authenticity (see Section 4.1).

- *We develop a working proof of concept (PoC) of a micro-payment-enabled over-the-top (OTT) platform to showcase IOTA value transactions.*

We have developed a new media streaming platform (OTT) based on pay-as-you-go and a consumption-based model that uses IOTA’s native cryptocurrency (iota). To the best of our knowledge, this is a kind of platform where the use directly pays the content creator, without any intermediary taking a profit share in the form of transaction fees (see Section 4.2 and Section 5.5).

- *The confirmation time of ‘value based transactions’ is reduced from linearly increasing time taking process to constant time (5.3 s) as average case using the index-based address value transaction (IBAVT) concept.*

Using the browser level storage capability, we stored the index of the address where all the balances are currently present to make it a stateful application. With this, we highly reduced the value-transaction confirmation time and overall latency (see Section 6.2).

- *Finally, several solutions are provided for the re-usable address issues persisting in the classical IOTA platform.*

Our work presents the readers with the state of the present classical IOTA platform maturity and discusses the weakness in the present system. We observed the problem of IOTA reusable addresses and have accumulated several solutions to solve this issue. We name this approach “reusable short address convenience” (RSAC) (see Section 6.4).

### 1.5. Paper Organization

The paper is divided into seven sections. The first section of the manuscript consists of an overview in the IoT ecosystem. It lays the foundation for the problems in the current IoT implementations and defines remedies for them through technological advancements in the Industry 4.0 era. The introduction discusses the implications of the shortcomings and the risk of ignorance behind the currently used methods. In the second section, a detailed description of the issues in the existing solutions of IoT ecosystem is provided. Along with security issues, it illustrates the state-of-the-art and provides insights into the historical background and research in the area of blockchain for the IoT ecosystem. Further, it provides insights about blockchain and the M2M economy. Section 3 describes each component of the IOTA platform beginning from the architectural level to different libraries. In Section 4, we describe how interactions and communications take place between devices and the IOTA Tangle. The section also introduces the mechanism of multiple protocols for secured communication and fully access control over shared IoT data in details. Section 5 focuses on the implementation fragment and multiple submodules of how results are achieved. It further highlights the key results achieved. Section 6 analyses and discusses several performance evaluation parameters like the storage management, scalability, transaction per seconds, implications, challenges, use, applications and security and privacy. This section holds the empirical evaluation of our paper in realistic view for the DLT for IoT adaptability and practicality for real world deployment purpose. The final section provides the future scope and concludes the manuscript.

## 2. Background and Related Works

The vivid and diverse aspects of the literature analysis done in the past present the idea how blockchain and DLT could fit in the modular space of the IoT. Moreover, facts from the past explain why studies on blockchain in IoT have seen a boost in academia as well as in the industry.

### 2.1. State of Art of Blockchain for IoT

Table 1 presents related works on blockchain for IoT under various focus areas.

#### 2.1.1. Access Control and Authentication Using Blockchain

Putra et al., used the Ethereum private network on Docker containers based on a trust and reputation system (TRS) for providing access to IoT devices [25]. However, their system results in noticeable delays in the latency achieved for real time IoT ecosystems.

Cha et al.’s protocol provides access management of IoT devices using a blockchain- connected gateway (BCG) serving as intermediary between IoT devices and users [26]. It is implemented with the help of BCG smart contract and device smart contract for privacy policies. However, if four attack vectors such as secret disclosure, replay, traceability and reuse token address are applied to the Cha et al. model, the success probability of the attacks reaches 1. Hence, an improved version of Cha et al., model was proposed in [27] that provides an improved blockchain-based authentication protocol (IBCbAP) for IoT network management using a hash-based message authentication Code (HMAC) signature mechanism. It is implemented in JavaScript and Ethereum local networks using the Web3 library and Test RPC. However, despite decent improvements compared to its predecessor, IBCbAP fails to handle the transfer of ownership of devices.

The authors in [1] proposed a decentralized micro-payment use-case between an electric-car and charging stations using DLT-based solution that enable a future smart devices economy via a simulation based on IOTA value channels. The proposed work also evaluated their solution on a smart home scenario where multiple consumer electronics can communicate with each other in a secure way.

#### 2.1.2. Security and Privacy Using Blockchain

In [28], Dorri et al. identified the implementation challenges of blockchain in IoT like latency, overheads, and bulkiness of the legacy blockchain mechanism. In order to mitigate these challenges, they proposed a light weight blockchain-based architecture without compromising on security and privacy issues. The core idea is to establish a distributed trust model which eliminates the processing time required for block validation. The proposed architecture was tested in a smart home network in a simulated environment. The results show the effectiveness of the proposal in mitigating the challenges of classical blockchain implementations and upholding the security and privacy benchmarks.

Further in [12], Dorri et al., provided an extension of work reported in [28]. Here the authors comprehensively describe the components of smart home tier out of the three-tiers identified in [28]. The role of a miner is explained in details with respect to smart homes. The simulation results indicate better mitigation of overheads and improved security.

Similarly, Zhou et al., highlighted the limitations of classical blockchain technology including computation costs, high bandwidth consumption and other implementation challenges. They proposed a new approach which uses blockchain and homomorphic computation approaches to service the requests of users [29]. The use of a homomorphic approaches makes it a privacy-preserved system where the system works on the data without knowing the actual data and thus preventing an attacker from learning from it. The proposed Beekeeper system was implemented on Ethereum platform for empirically evaluating its performance. The results indicate decent performance improvements.

In [30], Hassan et al., provided a discussion on the importance of privacy and security in blockchain-based IoT systems. The implementation challenges while integrating blockchain in legacy IoT systems are also discussed. Specific privacy concerns like anonymization, encryption and differential privacy have been covered in detail.

Rahulamathavan et al., provided an attribute-based encryption model for preserving the privacy of the users and devices in IoT ecosystems implemented using blockchain technology. The prime contribution includes providing controlled and limited access to the data among the participating entities requesting the use of data [31].

Le et al., provided a discussion about the importance of identity privacy with respect to forensics [32]. They proposed an identity-preserved blockchain based framework to enhance the confidentiality, integrity, and availability (CIA) properties of forensic evidence.

The model proposed in [33] describes an innovative use of blockchain and the analytic hierarchy process (AHP) to protect intruders from eavesdropping in the Industrial Internet of Things. However, the experimental result indicates that as the number of nodes increases, power draining will increase too.

#### 2.1.3. Blockchain-Based Applications

Dwivedi et al., acknowledged the role of IoT in the healthcare domain, specifically in terms of wearable technology and remote monitoring of patients [34]. The privacy and security issues related to healthcare big data were identified and a novel privacy preserving blockchain-based healthcare IoT system was proposed in the paper. The prime aspect of the proposal is how to effectively manage the medical big data through blockchain. For providing advanced security features, the proposal uses both symmetric and asymmetric cryptography for serving different purposes. A theoretical analysis was performed identifying the novel features of the proposed approach.

In [35], the authors provided a discussion on the role and applications of blockchain and IoT technologies in the food industry for tracing and tracking food throughout the production life cycle covering all entities involved in the agricultural ecosystem.

Rahman et al., provided a sharing economy concept using the integration of blockchain and IoT technology for a smart city ecosystem. Several examples like car rentals, healthcare services, location awareness and related services are discussed [36]. The proposed framework [37] makes use of a DLT-based vehicular public key infrastructure for a faster and intelligent sharing of services across the smart city ecosystem.

Valid use cases such as gaming, asset tracking, cryptocurrencies and intellectual property protection were empirically analyzed in [38] to find the best fit use case for blockchain. Similarly, Zavolokina et al. [39] discovered innovative solutions for digital car dossiers to increase trust and transparency and tracking the lifecycle of cars using a blockchain-based system.

In [40], Malik et al., provided a model for effective and trusted tracking and tracing of supply chain using blockchain-enabled IoT systems. The actors involved in the supply chain are rewarded through dynamically calculated trust and reputation scores based on their nature and type of interactions. Further a smart contract is created among the participating entities for effective and transparent collaboration among them.

#### 2.1.4. IoT Data Marketplace

Ahsan et al., have used the public Ethereum testbed as well as the private Hyperledger Fabric for secure and fast sharing of IoT data using proxy re-encryption schemes to form a trading platform. After analyzing the scalability issue, they used Hyperledger Fabric to test the performance with 25 TPS up to 200 TPS based on ‘invoke’ and ‘query calls’ [41]. However, due to the extensive reliance on Ethereum-based smart contracts, transaction confirmation delays were observed.

#### 2.1.5. Challenges and Limitation in Blockchain-Based IoT Applications

In [42], Alam analyzed several paradigms in the domain of blockchain for IoT to state the challenges and limitations of the domain. It projects several key challenges such as scalability, need for interoperability, security and privacy.

Panarello et al., provided an exhaustive survey of blockchain and its integration with the IoT ecosystem. Several aspects including access management, scalability, device management, implementation issues are discussed [43]. The paper also discusses blockchain application in a machine economy. Finally, the open research areas in seamless integration of blockchain with IoT are also discussed.

##### Need for Lightweight Clients for Blockchain

In [44,45,46], the authors proposed a lightweight scalable blockchain for the resource- constrained IoT ecosystem. Dorri et al., provide end to end security with an overlay network [47]. An overlay network is created in which computationally heavy devices manage the blockchain. The overlay is distributed into clusters to balance the load and reduce the overhead where the cluster head manages the public blockchain functions. The proposal in [48,49] introduces distributed throughput management to ensure even distribution of workloads for consistent throughput. The simulation results indicate a reduction in overheads and increased scalability.

##### Block and Fees Evaluation

Aldweesh et al., experimented with Ethereum fees with respect to opcode using OpBench on three different machines and two different clients, one in Go and other in Python. Their result shows a difference in the performance of Go clients and Python-Ethereum clients that make Ethereum less flexible and modular [50]. Also, fees moderation is not always proportional to the miners’ invested CPU time required to execute opcodes in the Ethereum Virtual Machine. Nepomuceno et al., described an innovative way of evaluating internet web pages according to load time, objects and size of web page to measure internet efficiency that could potentially see some usefulness in blockchain applications [51].

Sagirlar et al., worked on different networks based on the size of blocks, number of IoT devices and the device location was analyzed using Bitcoin Simulator. Their work presents that each parameter has a role to play in block generation, like block interval, device location, etc. [19]. However, the same scalability issue persists in the Ethereum network until the consensus mechanism is shifted from proof of work (PoW) to proof of stake (PoS) [5]. Although the maturity of the Medalla, the Ethereum 2.0 based on PoS is yet to evolve and be tested. The security, however, is naturally ensured using any blockchain architecture [40]. A better and highly reliable system is needed to act as next generation blockchain or the intelligent blockchain system for IoT. IOTA [7] is the next generation blockchain and would scale better for IoT applications as depicted in [52,53,54]. For the same, we have presented the IOTA-based architecture for IoT and a future M2M economy to deal with challenges of blockchain and IoT.

### 2.2. State of the Art of Blockchain for M2M Economy

Machine-to-machine (M2M) economy is the next step in the revolution of Industry 4.0 and technological evolution. With blockchain involved in the process of communication, M2M can be realized in efficient and secured manner [55]. The widespread use of devices and deployment lead to an urgency for secured communication for trust and intermediation in the vehicular systems [56,57,58,59]. Table 2 defines the state of the art of convergence of blockchain with M2M economy as a whole becoming an integral part of daily life.

The state of the art of development in the blockchain ecosystem hinders its wide adoptability due to low TPS, low confirmed transaction per second (CTPS), storage management, scalability, exposing smart contract vulnerabilities and heavy reliance on wallets, consensus protocols and miners [71,72]. Interoperability remains the weakest part of such architectures. Hence, our proposed technology and architecture is modular and built on top of the classical IOTA protocols, whereby no change in the traditional stack for interoperability is required.

### 2.3. Performance Parameters

In our research, we largely focus on two aspects i.e., evaluating the performance parameters of IoT applications and the other on security aspects. It includes full access control and ensuring data integrity via secured communication using DLT platforms. Hence, following performance criterions were taken into consideration that our study aims to provide definite answers for:

- Integration of IOTA with IoT devices (to check the performance parameters):
  Under this, we tested the following:
  1. Confirmed transactions per second (ctps)/throughput
  2. Latency
  3. Network bandwidth
  4. Size of network for scalability
  5. Energy consumption for low-power IoT devices
- Security: Setting up a secure communication medium using the masked authenticated messaging protocol
- Complexity: To check the overall implementation smoothness

## 3. Dissecting the IOTA Platform Ecosystem

IOTA in essence works significantly different than blockchain platforms currently available. In the following section, IOTA platform features and working are thoroughly explained.

### 3.1. Overview of the IOTA Platform

The IOTA Foundation was founded in 2015 by Serguie Popov, Dominik Scheiner, Sergey Ivancheglo, etc. By December 2015, the IOTA foundation had raised 1337 Bitcoin (approx. $0.5M) through initial coin offerings for project development (https://messari.io/asset/iota/profile accessed on 20 May 2021). The initial release of IOTA has been live since July 2016. Similar to the Satoshi (in Bitcoin) or Wei (in Ethereum), IOTA’s native cryptocurrency ‘iota’, is denoted as ‘i’ which is the smallest unit of the IOTA network.

#### Features of IOTA

The following are core features of the IOTA platform [7]:

(1) It offers decentralization. Any node can easily join the network or be part of consensus.
(2) Internally, the data structure used in IOTA is a directed acyclic graph (DAG) which in the IOTA ecosystem is called the ‘Tangle’.
(3) There are no transaction fees involved for miners. It is a miner-free platform where all IOTA tokens have been created.
(4) IOTA has deployed a post-quantum cryptography method which is based on a hash- based algorithm [73]. This particular algorithm is named the Winternitz One Time Signature scheme (W-OTS) [73].
(5) Each node is a validator. In this platform, before we submit our transactions, we have to validate two previous transactions of others [7]. This way as more new transactions will join the network, more previous transactions will get confirmed.

Micropayments were never possible in existing blockchain platforms due to the fact transaction fees were higher than the transactions themselves, but with the IOTA fee-less environment, micropayments can happen for the future M2M economy.

### 3.2. Architecture of IOTA Platform

The IOTA platform is similar to Ethereum blockchain but the overall architecture involves several distinguishing layers and components. This manuscript briefly explains all the components of the IOTA platform in sufficient depth. IOTA too consists of a node architecture having different client types, communication protocols to network types as in the case of Ethereum blockchain (such as Main Net and Test Net). In IOTA, the community network (Comnet) is mostly used for testing and development purposes and the IOTA Foundation development in this regard is completely transparent to the community as a whole. We have analyzed the proposed work (Section 4.1 and Section 4.2) in this study based on the Comnet network.

Like the Ethereum block structure, IOTA has a transaction/bundle structure for each transaction in the directed acyclic graph Tangle data structure. The basic building block of one transaction comprises a transaction hash, value, confirmation status, tag, address, bundle, nonce, signature message fragment and address of parent transaction that this transaction approves to.

**Figure 1:**
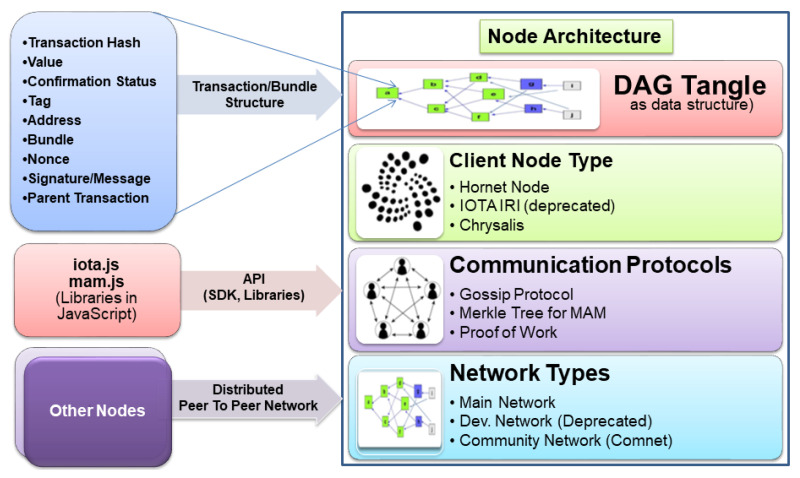
Architecture of the IOTA Platform.

Similar to the way *web3.js* is a client application programming interface (API) library in Ethereum (https://web3js.readthedocs.io/en/v1.3.0/, accessed on 20 January 2021), IOTA has provided *iota.js* and *mam.js* as client API libraries for building applications in JavaScript language. The architecture of IOTA is shown in Figure 1.

### 3.3. Components of IOTA Platform

**Figure 2:**
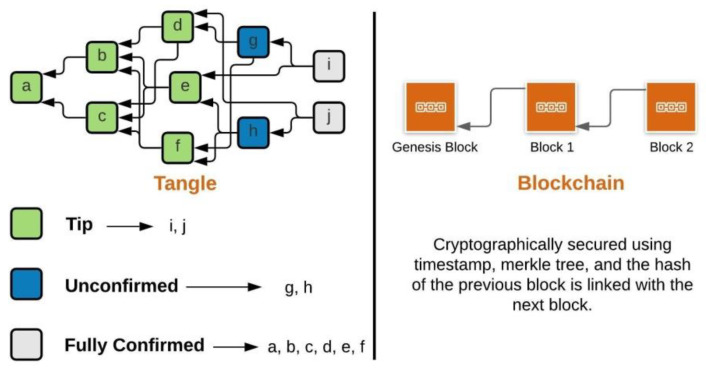
IOTA ‘Tangle’ versus Blockchain.

The boxes shown in Figure 2 are called transactions/bundles and consist of multiple transactions within them, analogous to the blocks in blockchain.

Bundles are treated as atomic, i.e., either all the transactions within a bundle get confirmed or none all together. In IOTA, tips are the newest transactions in the network as shown in Figure 2 and have not been referenced by any other transactions but they reference two previous transactions using the Markov chain Monte Carlo (MCMC) tip selection algorithm [7]. Fully confirmed transactions are transactions that can be referenced directly or indirectly by all the tips. Unconfirmed transactions are transactions that are referenced directly or indirectly by a subset of tips.

#### 3.3.1. Steps to Create a Bundle of Transactions for Attaching to the Tangle

The approach involves three fundamental processes for each bundle to be created by the user or an IoT device:

- *Transaction signing*: First, the node (computer/IoT device/application) prepares a valid transaction for sending it to the Tangle by signing it using the node private key.
- *Selecting two previous tips*: using the Markov chain Monte Carlo (MCMC) tip selection algorithm, the node analyzes the weighted mechanism and decides to choose two other previous transactions that are not confirmed yet [74]. Also, the tip selection must avoid the phase of being the lazy tip i.e., choosing two old transactions already confirmed by many others. Hence, coordinator is used for determining the tips using the tip selection API to ensure no one is selecting tips for their profit making or approving the double spending [75].
- *Proof of Work (PoW)*: the node must check two chosen tips to be conflict-free from double spending, etc., and then perform a level of proof of work against spam protection defined by minimum weight magnitude of the network.

#### 3.3.2. Transaction Confirmation

By definition, every confirmed transaction is one whose path exists from any of the tips in the network. Each tip must be directly or indirectly referenced to it. Hence, as more and more new tips will join the network, the transaction confirmations will increase. It further serves as a confidence parameter in deciding the confirmation rate for a tip to be selected. Although, as the depth size grows, it will result into longer validation and confirmation time. However, many other tip selection algorithms are proposed in RFCs#0008 i.e., weighted Uniform Random Tip Selection (https://blog.iota.org/the-tangle-an-illustrated-introduction-4d5eae6fe8d4/, accessed on 25 June 2019) (URTS) where were developed for better efficiency and higher confirmation rate [76].

#### 3.3.3. Client Nodes in IOTA

**Figure 3:**
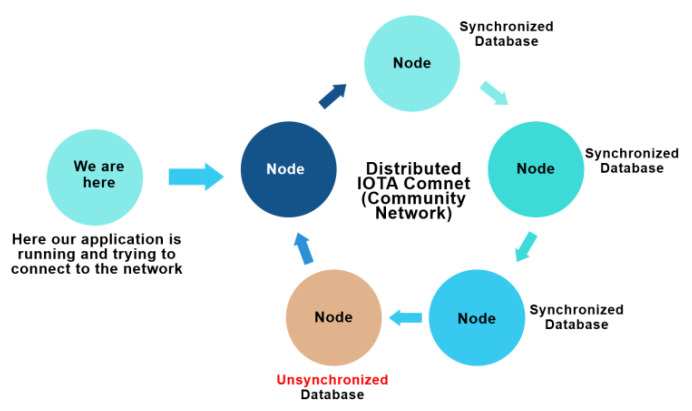
Network of IOTA nodes.

IOTA implemented IOTA Reference Implementation (IRI) as the first full node solution. However, this was negatively affected by to its heavy reliance on the computational power which is a key constraint in low power IoT devices. Figure 3 demonstrates how the network consisting of IOTA node is connected.

**Figure 4:**
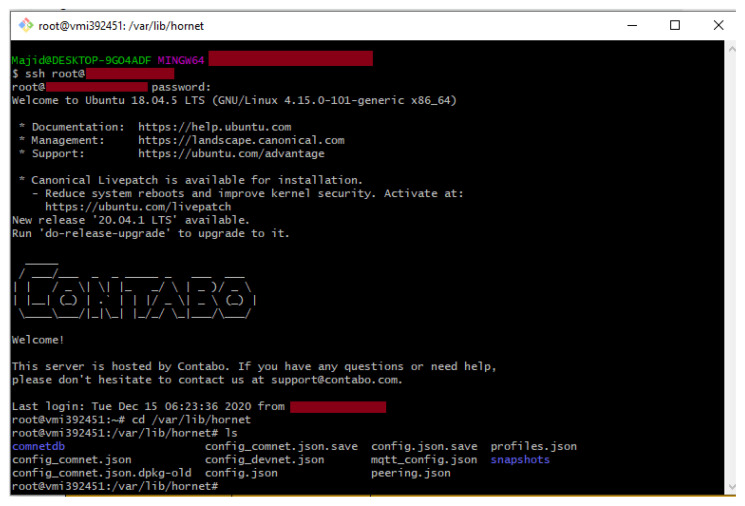
Accessing the Hornet node using SSH via Terminal.

Presently, Hornet is the official second full node after IOTA IRI for the IOT devices or the low power client devices that can even run under 300 MB of available RAM. Moreover, we installed the Hornet community network (Comnet) on the Ubuntu 18.04, VPS. We used *Contabo VPS* for the setup of a Hornet node and remotely accessed it using the SSH protocol via the Git-Bash terminal using command ‘*ssh root@<IPaddress>*’ and with our chosen ‘*password*’. The root folder of Hornet is under ‘*/var/lib/hornet*’ directory and the list of the files of the same directory is displayed in Figure 4.

Regularly, spam tests happen on the network from the Comnet community of the official IOTA Foundation to check its TPS and CTPS. These spam tests results led to better research around the platform and popular bug fixes.

Moreover, Tangle has a special property of getting ‘history’ branched out from the network based on how heavy a branch becomes or it even depends on the pruning index delay set by user/admin in the *config_comnet.json* file of the hornet node.

In the MCMC algorithm, a double spending event might still happen or some tips cannot get selected [77]. This problem cannot happen in a bigger network, but since at present, the IOTA network is small, comprising a few nodes, a ‘*Coordinator*’ (in short Coo) is placed as the supporting stem which keep a check on the activity of the transactions and prevent the double spending from happening. The network is still decentralized because each node can keep track of the activities of the coordinator to avoid approving double spending.

#### 3.3.4. Snapshot

Snapshot is used to reset Tangle when the size of ledger increases. It lowers devices’ burden of storing a complete history of the Tangle. It groups all the transaction balances into a new address and maintains only the final balance [7].

If a node wants to store everything without snapshotting or pruning, the solution is called a permanode which stores the entire Tangle securely. The official project for the permanode framework established by the IOTA Foundation is Chronicle (https://blog.iota.org/introducing-chronicle-a-permanode-solution-8e506a2e0813, accessed on 22 January 2020). The permanode concept is out of the scope in our paper. In Section 6.1.1, we have however devised a formula based on the number of days we want to store the Tangle data on our node. We will prune out rest of the history data from our node maintaining only the final updated ledger state.

#### 3.3.5. Hash Function: Kerl/Keccak-384

IOTA modified their ternary hash function Curl to the binary hash function Kerl (based on Keccak-384) after a vulnerability was found [78]. The Kerl hash function is used for generating the consecutive addresses from the seed and then using in signing transactions. This works similar to the sponge and squeeze function as described in the Keccak-384 core implementation.

#### 3.3.6. Trits and Trytes Alphabet

Primarily, with the recent advancement in the construction of chips and integrated circuits, IOTA had planned to utilize the ternary system (having three values) instead of binary system (having two values) on the first ever built ternary chips named a under project named JINN (https://iota-news.com/the-origins-of-jinn-and-iota/, accessed on 25 January 2020). Such ternary systems are either balanced $\left(-1,\;0,\;1\right)$ or unbalanced $\left(0,\;1,\;2\right).$ IOTA uses a balanced ternary system as software implementation emulated on binary hardware in its core libraries where a trit can possess values −1, 0 or 1. Trits in IOTA is analogous to bits and trytes is analogous to bytes (3 trits), for example −1 0 1 or 1 1 1 is a tryte. With this understanding, we can realize following ternary numeral system where [79]:

$$1\;Byte=2^{8}=256\;\mathrm{combinations}$$

$$1\;Tryte=3\;trits=3^{3}=27\;\mathrm{combinations}$$

##### IOTA Tryte Alphabet

**Figure 5:**
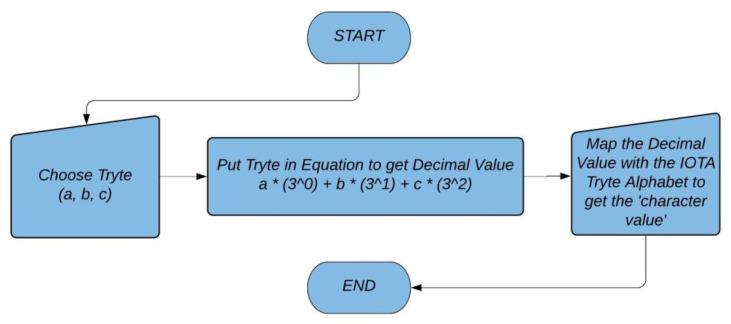
Flowchart for finding the Tryte Alphabet.

The Tryte Alphabet mapped with the ASCII character set are used in IOTA. However, character mapped with tryte can be determined based on the calculation used in the equation shown in flowchart diagram in Figure 5.

There is a conversion method by which the trits and trytes value look more human readable. This readability comes after the conversion done based on the Tryte Alphabet chart [79]. Also, an IOTA address once used in the signing process is not considered safe for another use. Hence, it is strictly advised not to reuse the same address twice for sending a transaction because a part of the arbitrary size of the key is revealed in the process of signing as IOTA uses the W-OTS signature scheme. However, one can receive as many transactions as wanted to a particular address, but once this address is used in sending some value from A to B, it is advised not to reuse this address again for receiving or sending further transactions. Hence, all hashes, address, seeds etc, are variants of trytes which are represented by combination of such tryte alphabet characters.

**Example** **1.** *ASCII text “Cat” will be “MBPCHD” in Trytes*.

**Proof of Example 1.** 
Since, ASCII value of ‘C’ is 67


67%27=13= M 
(from [79])
67/27=2= B 
(from [79])
Which means ‘C’= MB in tryte.
(1)


and since, ASCII value of ‘a’ is 97


97%27=16= P 
(from [79])
97/27=3= C 
(from [79])
Which means ‘a’= PC in tryte.
(2)


and since, ASCII value of ‘t’ is 116


116%27=8= H 
(from [79])
116/27=4= D 
(from [79])
Which means ‘t’= HD in tryte.
(3)
$\mathrm{Using}\;\mathrm{eqn}.\;\left(1\right),\;\left(2\right)\;\mathrm{and}\;\left(3\right),\;\mathrm{we}\;\mathrm{get},$


Hence, ‘Cat’= ‘MBPCHD’ in tryte

One can find more such conversions using the IOTA utility tool (https://asecuritysite.com/encryption/iota, accessed on 20 January 2020). □

#### 3.3.7. IOTA Seed and Addresses

**Figure 6:**
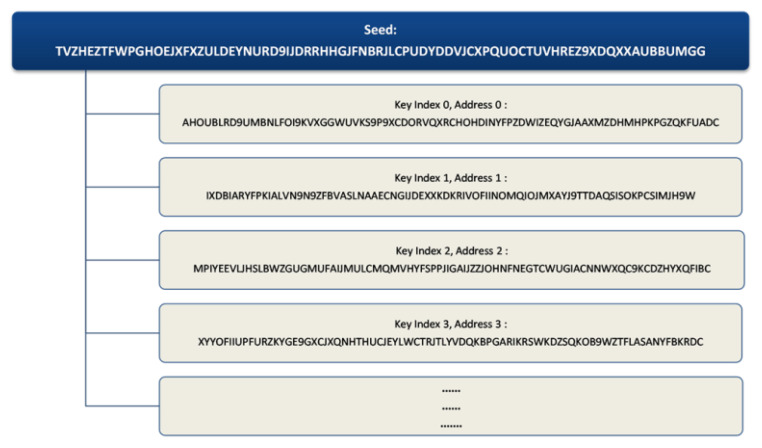
Address generation from seeds.

An IOTA seed contains combination of 81 uppercase characters which result to 81 trytes [7]. Each tryte has 27 combinations, as shown in Section 3.3.6, which means an IOTA seed has 27^81^ ≈ 8.71 × 10^115^ combinations and on the other hand, a bitcoin random number has 2256 ≈ 1.15 × 10^77^ combinations [79]. In a UNIX or Linux environment, the command ‘*cat/dev/urandom |tr -dc A-Z9|head -c${1:-81}*’ will produce the 81 characters of a required unique seed using the entropy pool collected by the mechanical properties of devices and current timestamp (https://www.iotaprice.com/strong-seed-iota.html, accessed on 20 June 2020). With this unique seed at our disposal, we can generate corresponding addresses using the key index number. Example, address 0 has key index number 0, address 1 has key index number 1, etc., as shown in Figure 6.

On creating an address, we can specify a ‘security level’ ranging from 1 to 3. This indicates how long we want our private key and signature fragment to be in trytes. Security level 1 has 2187 trytes, security level 2 has 4374 trytes and security level 3 has 6561 trytes of private key and signature length. In practice, security level 2 is used by default.

#### 3.3.8. Minimum Weight Magnitude (MWM)

MWM is used for the IOTA’s proof of work (PoW) just like Hashcash in Ethereum and Bitcoin for the number of difficulty level decided by the trailing Zeroes.

*For Mainnet*: 14
*For Comnet*: 10

The PoW in the IOTA network is comparatively much lower in computation compared to the PoW in Ethereum and Bitcoin. Further, if an IoT device does not want to perform PoW at their device end due to constraints, then they can opt for third party remote PoW or set-up their own node end for the remote PoW computation. In our study, we have enabled the remote PoW at our node to perform PoW on behalf of IoT devices.

## 4. Methodology

**Figure 7:**
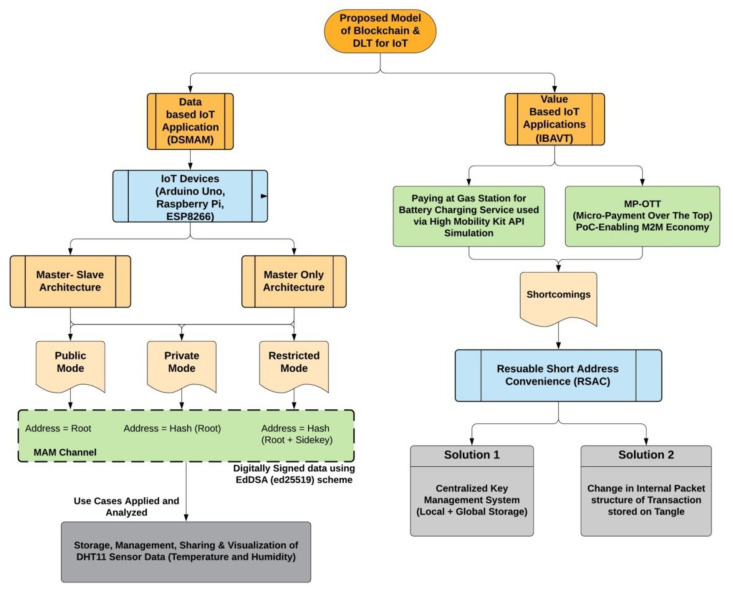
Proposed Architecture of DLT for IoT.

The proposed architecture is divided into two separate channels, i.e., Data and Value, as shown in Figure 7.

### 4.1. Data (IOTA Data Based System)

The data part will work on the IoT devices (in our case-Arduino Uno, ESP8266 and Raspberry pi 3b) for acquiring sensor data from a DHT11 (digital humidity and temperature sensor module) from the environment and send this data to the Tangle. It is further divided into two infrastructural architectures, i.e., master-slave architecture and master only architecture. IoT devices like Raspberry Pi acting as master nodes (MNs) are capable of processing data from the sensor attached to them and then sending this data to the Hornet node (HN) using IEEE 802.11n (Wi-Fi) as exchange protocol messages for further attaching it to the Tangle. However, other IoT devices are not capable of doing the same, hence, as self-acting slave nodes (SN), they will process the data from the sensors and then forward this data packet to the nearest MN either through IEEE 802.11n, Bluetooth or Arduino serial communication protocol responsible for sending to HN.

Based on the merkle tree root, a data is sent to the Tangle signed by the private key using the Kerl hash function (a variant of Keccak-384) (https://github.com/iotaledger/kerl, accessed on 10 June 2020) to prove the authenticity of the data to the network, but this signing action does not ensure that data is actually generated by the trusted IoT device and no man in the middle attack happened, as we cannot share the seed with anyone. However, after sending data to the Tangle, data is propagated to the whole network (to all nodes) using the Gossip protocol. All the data stored with merkle roots are connected and linked with each another resulting in a forward chain. Each root stores the data and references the next merkle root. This way, each root can decode the message in the next root maintaining a forward secrecy, but no root can look behind in the line. Anyone having a root can decode the whole channel message afterwards from the Tangle. Furthermore, to gain the access control over the data, three mode/channels for sending the data packet to the Tangle are described, namely public mode, private mode and restricted mode. These channels are known as masked authenticated messaging (MAM) channels. In practice, addresses are the actual placeholder where data is stored and can be accessed with. In public mode, no data encryption is done, hence the address is simply the root. In private mode, encryption is applied and the address is the hash of the root. In restricted mode, a *sideKey* is incorporated to press revoke/grant access to the channel. Here, address is the hash of the root and sideKey (https://medium.com/coinmonks/iota-mam-eloquently-explained-d7505863b413, accessed on 10 February 2020).

- *Public Mode Channel*
  address=root

- *Private Mode Channel*
  $$address=Hash\left(root\right)$$

(MAM message is read using root).

- *Restricted Mode Channel*
  $$address=Hash\left(root+sideKey\right)$$

(MAM message is read using root and sideKey).

To make sure there are no man in the middle attacks, and data is actually being generated by the trusted IoT device, we propose dual signature masked authenticated message (DSMAM) by introducing one more level of signing of the IoT data packets using the (EdDSA), which is a new signature scheme based on the Schnorr signature algorithm and Elliptic curve. Internally, the EdDSA algorithm relies on (Ed25519) signature based on Curve25519 and SHA-512/256 to ensure the authenticity and the data integrity of the IoT device generated data. Our algorithms in DSMAM for key generation, signing and verifying for IoT devices are Algorithms 1–3 respectively.


**Algorithm 1. Key generation of IoT devices**
Step 1:**for** each IoT device **do**Step 2:  generate keys using EdDSA (ed25519) schemeStep 3:  export publicKey, privateKey in ‘PEM’ formatStep 4:  generate Seed of 81 Trytes characterStep 5:  embed privateKey and Seed in IoT deviceStep 6:
**end for**



**Algorithm 2. Signing of data packets**
Input:mode, sideKey, privateKey and interval
*Initialization*
:
Step 1:connect to synced node endpoint urlStep 2:calculate first root using seed for sending data

*LOOP process with* (*root*, *payload*, *interval*) Step 3:**for** every interval **do**Step 4:   receive data from sensors to master node *temp* = sensor temperature data *humd* = sensor humidity dataStep 5:   create message payload *payload* = (dateTime, temp, humd)Step 6:  sign the payload using privateKey *signature* = sign(payload) Step 7:   store the signature in the payload *edsignature* = signature *newpayload* = (dateTime, temp, humd, edsignature)Step 8:   attach the new message payload to the Tangle attachToTangle(mode, sideKey, newpayload) *print*(*payload*, *address*, *root*, *nextroot*)Step 9:  Now REPEAT with root = nextrootStep 10:
**end for**



**Algorithm 3. Verifying of data packets**
Input:mode, sideKey, root and publicKey received
*Initialization*
:
Step 1:connect to synced node endpoint urlStep 2:fetch the first packet from the Tangle using rootStep 3:*flag* == true and *verified* == false

*LOOP process with* (*root*, *sideKey*, *mode*) Step 4:**for** each packet fetched from Tangle **do**Step 5:  **if** (*flag* = = true) **then**
Step 6:   get the first payload & retrieve signature *verified* = verify (Hash(payload), signature) *flag* = falseStep 7:  **end if**Step 8:   **if** (*verified* = = true) **then**Step 9:   fetch all rest of the packetsStep 10:  **else**Step 11:   returnStep 12:  **end if**Step 13:
**end for**


### 4.2. Value (IOTA Payment-Based System)

IOTA address plays an important role on how it is generated. Since the W-OTS scheme is used, each address must be used only once. However, transactions on IOTA are feeless, which enables the domain for the M2M economy, where multiple parties or devices can share information/services and get charged for the service they used in terms of micro-payments. To illustrate this power of M2M communication, we implemented the IOTA payment module in the novel proof of concept for the micro-payment enabled over the top (MP-OTT) media streaming platform service where the revenue model is a pay-as-you-go model. Content viewers will be charged based on the amount of time (in seconds) they watched the content at our platform and not according to the traditional way of subscription to media streaming platforms. We identified the classical IOTA value transaction payment consuming time in linearly increasing fashion and hence we propose an efficient constant time taking index-based address value transaction (IBAVT).

## 5. Implementation of Proposed Approach

The comprehensive implementation details are described in the following sections ranging from node setup to our developed DApps.

### 5.1. Setup and Arrangement of Hardware

**Figure 8:**
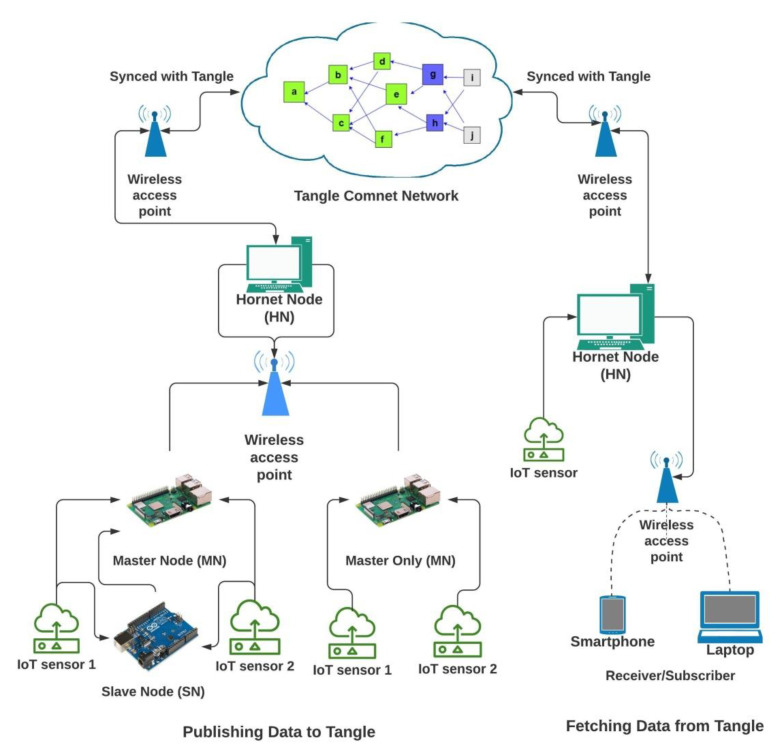
Setup of Hardware infrastructure.

The setup and arrangement for sending and receiving IoT data is shown in the Figure 8. This flow chart explores the interactive way how the hardware arrangement is set up. We implemented the proposed architecture following the system specifications and using the software shown in Table 3.

### 5.2. Process of Sharing of IoT Data

**Figure 9:**
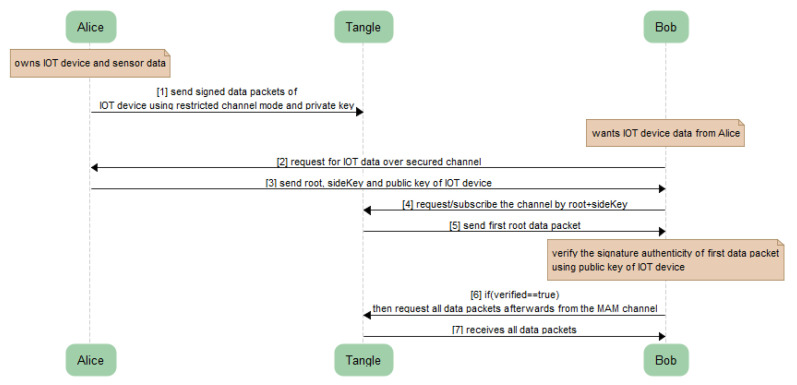
Sequence diagram for secured communication.

Users can securely and privately share messages with each other using the MAM channel described in Section 4.1. The communication can take place using the distribution of three input parameters ‘*Root*’, ‘*Public Key of IoT device*’ and a ‘*SideKey*’ (only if private and restricted modes are used, as shown in Figure 7). The receiver can then fetch the data/message payload from the respective MAM channel using our Algorithm 3 described above. Only the valid receiver having the correct combination of these three input parameters can fetch and decode the message in the encrypted packet of MAM payload. However, before fetching the entire channel data, the receiver can check the verification status of the first payload and if the resultant is found to be true and valid, the rest of the payload extant in the channel can be fetched and read. The sequence diagram for the secured communication between Alice and Bob will take place as shown in Figure 9.

### 5.3. Sending and Receiving Data

IOTA provides its client side libraries written in JavaScript namely *iota.js* (https://github.com/iotaledger/iota.js/, accessed on 10 May 2020) and *mam.js* (https://github.com/iotaledger/mam.js, accessed on 15 May 2020). However, the library *mam.js* might cause some errors when running on different machines. To prevent that, we regenerated or rebuilt the *mam.js* file using Browserify (http://browserify.org/, accessed on 13 August 2020) to create one single bundle file for *mam.js* to interact with web-browsers. In order to achieve that we first installed the browserify node module and *mam.js* node module. Then we created a *mamtobrowser.js* file with “*global.mam = require(‘@iota/mam.js’)*;” in it and run a command as “*node_modules/browserify/bin/cmd.js mamtobrowser.js --standalone window > mamweb.js*” in the console to get the *mamweb.js* file in the same directory. After that we can easily import it to our html page with “*<script src=“mamweb.js”></script>*” and use the MAM functions with “*mam.*”.

With the help of these libraries, we were able to build a command line interface application written in NodeJs for sending DHT11 sensor module data connected to an Arduino UNO and Raspberry Pi 3B model and receiving the data back from the IOTA Tangle.

**Figure 10:**
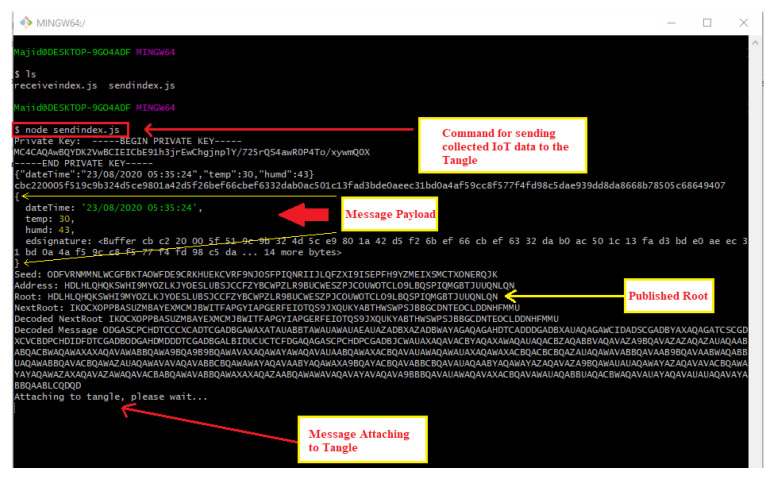
Attaching the first message payload to the Tangle.

Figure 10 displays the command line interface where a DHT 11 temperature and humidity sensor data is sent to the Tangle for intermediate decentralized storage using the function ‘*sendTrytes()*’ from the core API libraries. Before attaching data to the Tangle, the private key of the IoT device is used for generating and attaching a signature in the message payload for verification and authenticity.

In similar fashion, the application will keep on attaching new message payload (IoT data, such as temperature and humidity) to the tangle on a continuous interval of 5 s which we predefined in our application. 

**Figure 11:**
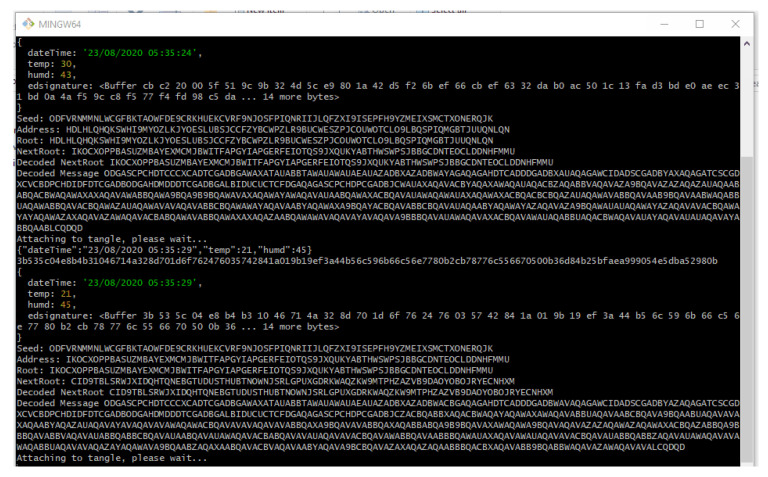
Attaching the rest of the message payload to the Tangle.

**Figure 12:**
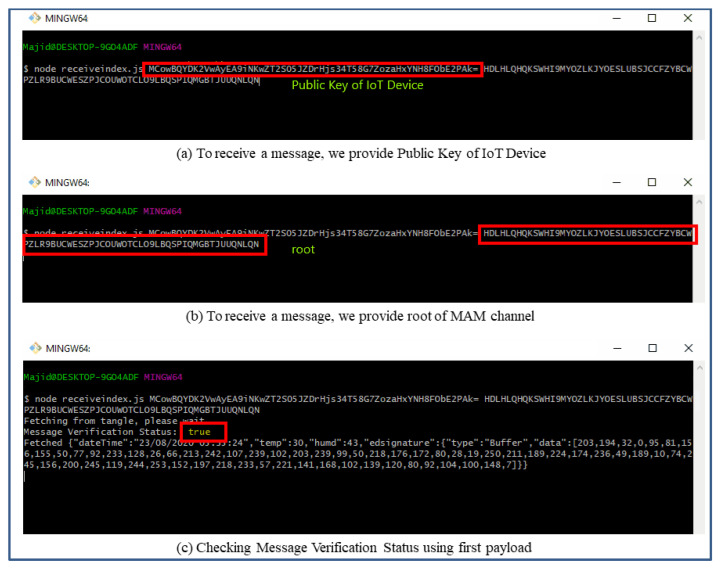
Fetching of the first message payload using a public key and root in ‘public’ mode.

**Figure 13:**
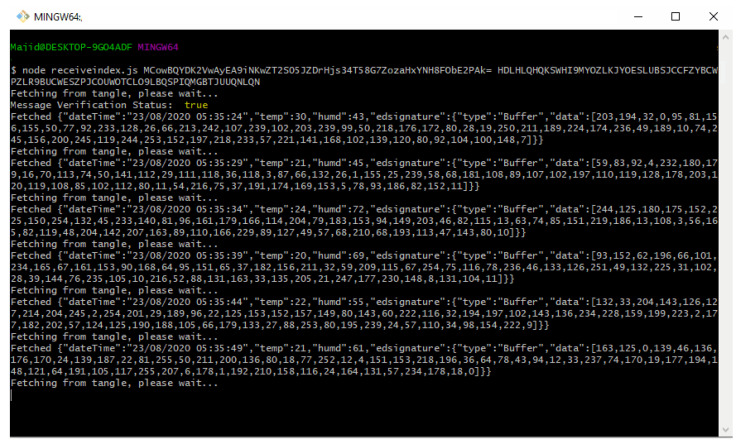
Fetching the rest of the message payload using a ‘public key’ and ‘root’ in ‘public’ mode.

However, the interval choice is based on the user and the requirements of any actual application. Figure 11 shows the attachment of a continuous epoch of payload to the Tangle in similar approach. Once all payload and packets are attached to the tangle, receiving the payload from the tangle becomes easy. According to our Algorithm 3 and the sequence diagram shown in Figure 9, we only need to fetch the first payload from the Tangle to check its validity that it is coming from the right person we had asked IoT sensor data from. To check the authenticity of the message payload, we need the ‘*public key*’ of the IoT device (the DHT 11 sensor in our case). Hence, we first verify the signature in the message payload of the first fetched data from the tangle by hashing the message payload (dateTime, temp, humd) as shown in Figure 11 and then comparing with the signature (edsignature) present in the payload itself. A verification message will be revealed as shown in Figure 12. By this virtue, if both signatures are found to be equal, a ‘true’ verification message will be displayed and it will fetch all the rest of the data packets extant in the MAM channel as shown in Figure 13.

### 5.4. IoT Sensor Data Visualizer App

**Figure 14:**
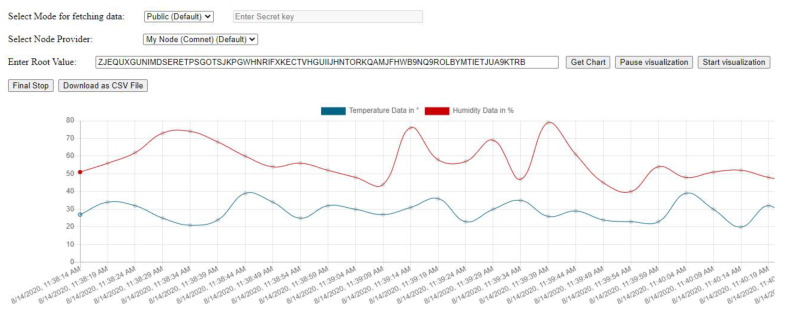
IoT sensor data visualizer app.

While implementing all this, we built a IoT sensor data visualizer application shown in Figure 14, where a temperature and humidity graph can be shown on the dashboard which can help ML Scientist or individuals better understand sensor data fetched directly from the Tangle.

A user has to provide the ‘*root*’ value of the MAM message to fetch the temperature and humidity data. This graph helps in determining the factual representation of the condition of the goods and items carried for which DHT11 sensor module is used for. Once, a user gives the *root* value, all the messages in the next of the MAM channel are fetched and displayed. Any anomaly in the value of temperature and humidity can be easily detected.

**Figure 15:**
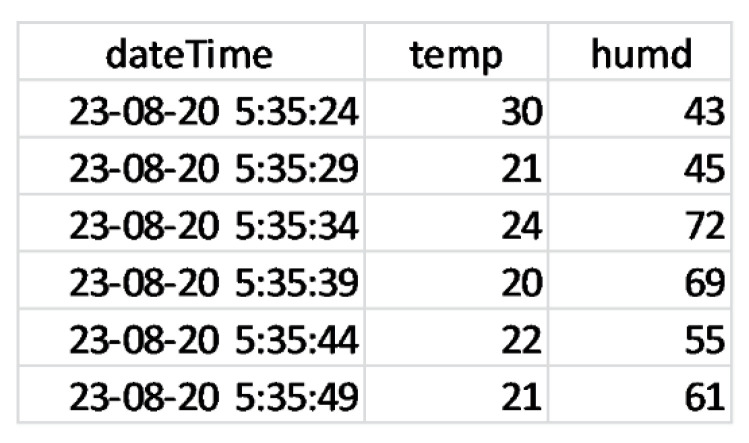
IoT data saved in a .csv file.

We also incorporated a facility for downloading the fetched data in to the ‘*csv*’ format as shown in Figure 15 based on the fetched data in Figure 13 to be useful for other platforms like Jupyter notebook for running advance data mining algorithms on the data.

### 5.5. Proof of Concept (PoC) For Micro-Payment Enabled over the Top (MP-OTT) Platform

**Figure 16:**
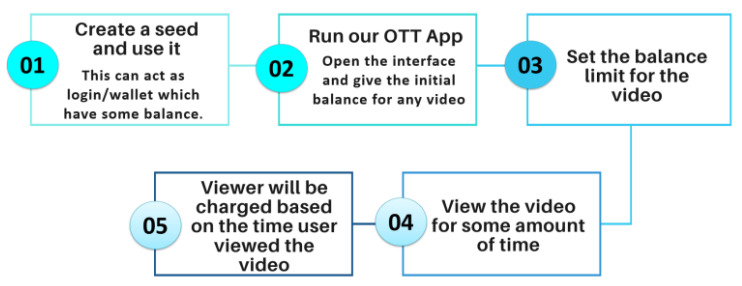
Transaction flow in PoC of MP-OTT.

The solution developed for the micro-payment opens a wide variety of applications and revenue models for a future M2M economy. In this novel PoC, a user is charged based on the time particular video content is viewed, following the “pay-as-you-go model” and “consumption based model”. This provides a fair platform to the content creators. The transaction or the payment is received directly to the content creator wallet in fashion analogous to peer-to-peer protocol eliminating the intermediaries. The transaction flow for the process used in the proof of concept of MP-OTT is shown in Figure 16.

**Figure 17:**
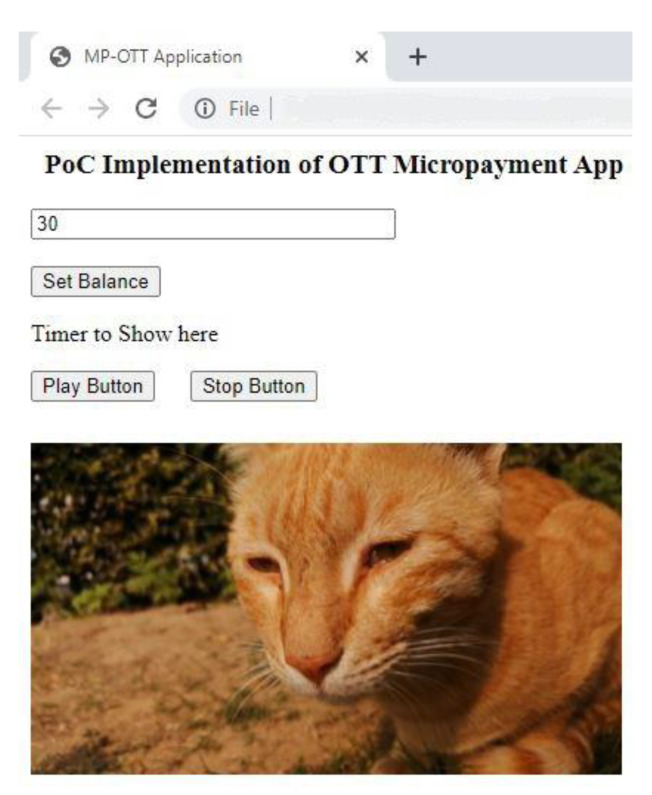
MP-OTT platform dashboard.

The front-end of the proof of concept OTT application is shown in Figure 17.

For validation of our work, we assume a user is watching a video on our OTT application. The moment the consumer clicks the stop button after 5 s, 5 iotas will be deducted from his account and they directly reach to the account of the content creator using our IBAVT. This payment is done in background and can be explored using the transaction link received back.

**Figure 18:**
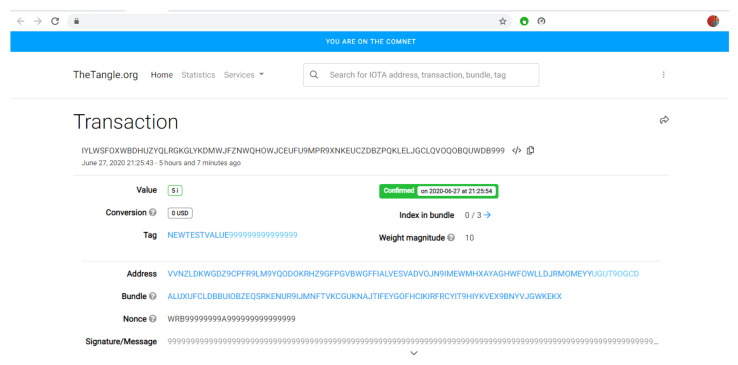
IOTA’s payment shown on website of comnet.thetangle.org.

If we click on the link in the transaction id, it will redirect to the Comnet Tangle Explorer official website (https://comnet.thetangle.org/, accessed on 25 August 2020). The webpage displayed on the Comnet Explorer using the transaction id received back is shown in Figure 18.

## 6. Results and Discussion

Analysis, discussion and the empirical evaluations which show significant performance improvements based on storage management, scalability, transaction per seconds, implications, challenges, use, applications and security and privacy are presented below.

### 6.1. Scalability and Storage

We found MAM channels to be an efficient way of communication, storing, managing and sharing IoT sensor’s data due to the following:

- *Scalability*: Scalability in terms of CTPS is set to increase with more IoT devices connecting to network as discussed in Section 3.3.2. These communications can be used for industrial IoT or commercial IoT applications.
- *Storage*: Storing IoT data on VPS will increase the size of data with time. We can set options for how long we want to store the IoT device data in the field pruning index in comnet_config.json setup file as discussed in Section 3.3.3. The node will prune the old data in the database according to the pruning index delay value set.

#### 6.1.1. Pruning Index Delay Formula

There are two methods to calculate and verify the pruning index interval value.

*Method 1*: Using number of days, we can directly calculate the size of database based on needs and resources available (after 30 days in this example). This method is approximate and not reliable due to changes in the actual TPS, but this can give an estimated size of the database if we take 30 TPS as average and 2673 Trytes as size of one transaction (https://domschiener.gitbooks.io/iota-guide/content/chapter1/transactions-and-bundles.html, accessed on 20 September 2020).

(1) $$\mathrm{Size}\;\mathrm{of}\;\mathrm{one}\;\mathrm{Transaction}\;\left(\mathrm{approx}\right)\mathrm{in}\;a\;\mathrm{Bundle}\;\left(\mathrm{TS}\right)\;=\;2673\;\mathrm{Trytes}\;\;\approx \;1800\;\mathrm{Bytes}\;\left(1600+\mathrm{Metadata}\right)$$

(2) $$\mathrm{Average}\;\mathrm{Transactions}\;\mathrm{per}\;\mathrm{Second}\;\left(\mathrm{AvgTPS}\right)\;=\;30$$

(3) $$\mathrm{Number}\;\mathrm{of}\;\mathrm{Seconds}\;\mathrm{in}\;a\;\mathrm{day}\;\left(\mathrm{TotalSeconds}\right)=60*60*24=\text{86,400}$$

(4) $$\mathrm{Number}\;\mathrm{of}\;\mathrm{Days}\;\mathrm{we}\;\mathrm{want}\;\mathrm{to}\;\mathrm{store}\;\mathrm{IoT}\;\mathrm{Data}\;\left(\mathrm{Days}\right)\;=\;30$$

$$\mathrm{Using}\;\mathrm{eqn}.\;\left(1\right),\;\left(2\right),\;\left(3\right)\;\mathrm{and}\;\left(4\right),\;\mathrm{we}\;\mathrm{get},$$

(5) Total size of transaction in Bytes = TS × AvgTPS × TotalSeconds × Days=1800 × 30 × 86,400 × 30=139,968,000,000 Bytes

(6) $$\mathrm{Approximate}\;\mathrm{Size}\;\mathrm{of}\;\mathrm{Database}\;\mathrm{in}\;\mathrm{GB}\;\mathrm{after}\;30\;\mathrm{days}\;\left(\mathrm{DbSize}\right)\;=\text{139,968,000,000}/1024\;\approx \;\text{136,687,500}\;\mathrm{KB}\;\;\;\;\;\;\;\;\;=\text{136,687,500}/1024\;\approx \;\text{133,483.88}\;\mathrm{MB}\;\;\;\;\;\;\;\;\;\;\;\;=\text{133,483.88}/1024\;\approx \;130.355\;\mathrm{GB}$$

(7) $$\mathrm{Number}\;\mathrm{of}\;\mathrm{Milestones}\;\mathrm{in}\;1\;\mathrm{Day}\;\left(\mathrm{MS}\right)\;\;\;\;\;\;\;\;\;\;\;\;=\;\mathrm{Number}\;\mathrm{of}\;\mathrm{Seconds}\;\mathrm{in}\;a\;\mathrm{day}\;/\;10\;\sec\;\mathrm{per}\;\mathrm{milestone}\;\;\;=\;\left(60\;\times \;60\;\times \;24\right)/10=8640$$

(8) Number of Milestones for 30 Days = MS ×30=259,200

Hence, ‘295,200′ is to be specified in the pruning index in the *config_comnet.json* file for storing data for a month (30 days) that can lead to approx. 130.355 GB.

*Method 2*: This is a heuristic method in which milestones (MS) can be directly manipulated to calculate the size of database. On using a certain MS value in the pruning index, if the resultant size is sufficient to handle for the node maintainer, then they can continue using the same MS as the pruning index for the Comnet Tangle.

(9) $$\mathrm{Number}\;\mathrm{of}\;\mathrm{Milestones}\;\left(\mathrm{MS}\right)\;=\text{259,200}$$

(10) $$\mathrm{Interval}\;\mathrm{of}\;\mathrm{Milestone}\;\left(\mathrm{interval}\right)\;\mathrm{in}\;\mathrm{seconds}\;=10$$

(11) $$\mathrm{Size}\;\mathrm{of}\;1\;\mathrm{Transaction}\;\mathrm{in}\;\mathrm{bytes}\;\left(\mathrm{sizeT}\right)\;=1800$$

(12) $$\mathrm{Average}\;\mathrm{Transaction}\;\mathrm{per}\;\mathrm{Second}\;\left(\mathrm{AvgTPS}\right)\;=30$$

$$\mathrm{Using}\;\mathrm{eqn}.\;\left(1\right),\;\left(2\right),\;\left(3\right)\;\mathrm{and}\;\left(4\right),\;\mathrm{we}\;\mathrm{get}$$

(13) $$\mathrm{Size}\;\mathrm{of}\;\mathrm{Database}\;\left(\mathrm{DbSize}\right)\mathrm{in}\;\mathrm{Bytes}\;=\;\mathrm{MS}\;\times \;\mathrm{interval}\;\times \;\mathrm{sizeT}\;\times \;\mathrm{AvgTPS}\;\;=\text{259,200}\;\times \;10\;\times \;1800\;\times \;30\;\approx \;\text{139,968,000,000}\;\mathrm{Bytes}$$

(14) $$\mathrm{Size}\;\mathrm{of}\;\mathrm{Database}\;\mathrm{in}\;\mathrm{GB}\;=\frac{\mathrm{DbSize}}{\text{1,000,000,000}}\approx \text{139,968,000,000}/\text{1,073,741,824}\approx \;130.355\;\mathrm{GB}$$

Hence, ‘295,200′ is to be specified in the pruning index in the *config_comnet.json* file for storing 130.355 GB data.

### 6.2. Performance Evaluation

#### 6.2.1. Efficiency Obtained in Term of Fast Payment Process in IBAVT against Classical IOTA Value Transaction Library

**Figure 19:**
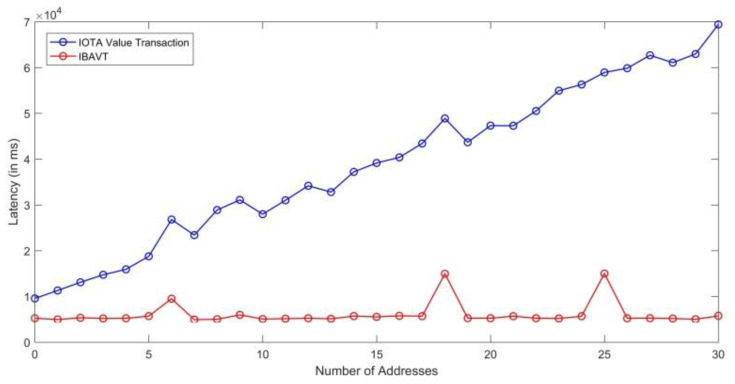
Comparison of latency between our proposed IBAVT and classical IOTA value transactions.

The IOTA current payment module works by finding balances at each address before making a transaction from person A to person B. We tested the IOTA payment library with the first 30 addresses where a balance is only present in the last n-th address. The library initially checks the balance in 1st, 2nd, 3rd addresses, ultimately reaching the n-th address which is a linearly increasing time-consuming process. This method is not efficient even if we only have 30 addresses with some balances in each. One seed alone can produce trillions of addresses. Hence, we transformed the application into an efficient and robust application using the browser level storage capability. We stored the value of index of the last address where all the balances are currently present and hence named this as index-based address value transaction (IBAVT). In IBAVT, we assume all the balances are present at a single address. Whenever, a new transaction is to be made, only the present index address where all the balances are currently present is checked and the transaction is made. This method highly reduced the value-transaction confirmation time to 5.3 s (5341 ms) as average case as shown in Table 4 against the linear time taken by IOTA library as shown in Figure 19 in the MP-OTT application for paying for each time a user consumed the content by viewing it. Our proposed IBAVT performed efficiently and fast for the communication of value transactions even if the addresses reach a high value. However, there are multiple ways to make a stateful application and other practitioner may adopt other technique to make a stateful application. With more addresses, the classical IOTA value transaction library will keep on increasing linearly.

#### 6.2.2. TPS Versus Confirmation Rate of the Comnet

**Figure 20:**
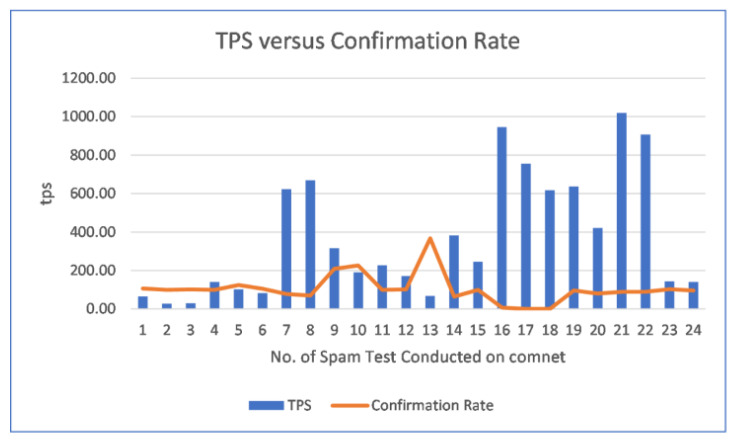
TPS versus Confirmation Rate.

We observed 24 instance of ‘Community Spam test’ from our Hornet node where multiple spammers joined using the official IOTA Foundation Discord channel arranged on 8th August 2020 and conducted spam tests using the ‘*luca-moser iota-spammer*’ program to check the Comnet capability for CTPS with respect to TPS (https://blog.iota.org/dev-status-update-august-2020-b08ccfd6f272/, accessed on 10 September 2020). The capability of the Comnet Network is shown in Figure 20 based on the spam test results in Table 5.

It is clear in the Figure 20, initially, when the spamming and TPS is low, the confirmation rate is high and over 100%. As spamming gradually increases, TPS increases and the confirmation rate start to decline at first. At the observed spam test number 16, when the TPS is close to 1000, the node went offline resulting in a node crash of the system which our VPS couldn’t handle. For the consecutive observed spam tests 17 and 18, the confirmation rate achieved was 0 thereafter at the out node. After, restarting the Hornet node, we joined the on-going spam test number 19 and onwards with once again observing high TPS crossing at 600 TPS and this time the confirmation rate at node end was found to be 96%. However, reaching over 1000 TPS resulted in a 89% confirmation rate. At the spam tests 23 and 24, spamming was brought to normal condition around 140 TPS achieving 96% CTPS.

#### 6.2.3. Advantage over Other Blockchain Platforms

Since the proposed architecture is miner-free and fee-less, it prevents the grouping of mining pools and domination of the network. In Ethereum, mining pools like Spark Pool, F2Pool or Ethermine control the mining process monopolizing in mining stakes. Recently, a man paid $2.6 Million as transaction fees to send $130 of ether [80]. Hence, it is not safer to promote IoT applications on top of such architecture. IoT systems must be protected from such malicious behaviors of nodes or participants.

#### 6.2.4. Performance Comparison with Other State-of-the-Art Work

For performance comparison, the proposed architecture is compared with seven related studies based on performance factors like scalability, energy consumption, confirmed transaction per second, reduced overhead/complexity, signature scheme, data integrity, access control, fee-less environment, time critical computing, decentralized nature and interoperability/modularity. The ‘✘’ corresponds the low performance and the ‘✓’ corresponds an acceptable performance for the respective category. It is observed in Table 6 that our proposed architecture with dual layer digital signature scheme in the classical MAM version 0.x (v0) provides all the necessary performance assurances.

### 6.3. Security and Privacy

We used the Edwards-curve digital signature scheme (EdDSA) for the second layer of signature module for proving the authenticity and data integrity of the data from IoT devices. EdDSA relies on Ed25519 which is newest and secured signature scheme used in cryptography. Since most of the earlier approaches used common signature methods like NIST P-254 and seckp256k1 they are not considered safe for use based on the SafeCurves (choosing safe curves for elliptic-curve cryptography) [81]. Ed25519 has now been adopted by the TLS 1.3, OpenSSH, saltpack, OpenBSD, GnuPG, cryptocurrency protocol and by many other softwares (https://ianix.com/pub/ed25519-deployment.html, accessed on 10 December 2020). Ed25519 provides speed benefits and security benefits. It is even immune to side channel attacks. Table 7 deliver some of the notable features of Ed25519 signature scheme over others based on work of Bernstein on Ed25519 Crypto [81].

#### 6.3.1. Non-Reliability of the Random Number Generator

Since all elliptic curves are based on random number generators which are calculated by the entropy, enjoined by the device drivers, mechanical delays, user interrupts, network traffic, etc. This ensures the true random number to be generated each time. However, IoT devices are not heavily equipped with mechanical hardware, hence calculating entropy won’t always result in true randomness, whereas, the EdDSA scheme doesn’t uses a random number generator and instead uses a deterministic way of generating signatures.

#### 6.3.2. Key Size, Signature Size and Payload Size

Since elliptic curves signatures yield small key sizes, hence they are very useful instead of RSA for achieving the same level of security. This helps in sending light data packets to the Tangle as the signatures are generated using small keys. The resultant signature using Ed25519 is only 64 bytes in size. This helps in keeping the payload size small.

#### 6.3.3. Fast Signing and Verifying

Ed25519 based on EdDSA provides the fastest signing and verifying functions [82]. Hence, this improves the overall user-experience for the IoT system without any overhead delay in the operations between data packets.

#### 6.3.4. Ensuring the Same Performance of DSMAM against IOTA MAM Protocol Along with Enhanced Security

**Figure 21:**
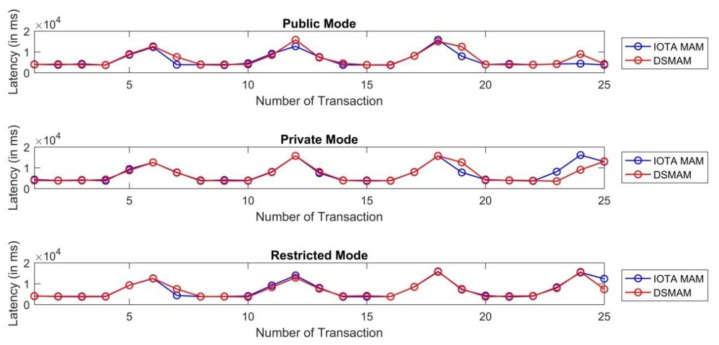
Comparison of latency between IOTA MAM and our proposed DSMAM.

We used the Ed25519 signature algorithm on the existing IOTA MAM channel to ensure the authenticity of each message based on the key pairs of IoT devices. We named this double signature implementation on MAM channels as Dual signature masked authenticated message (DSMAM) and have conducted empirical evaluations based on the latency achieved before and after our implementation as shown in Figure 21. The test involved 25 transactions at an interval of 10 s based on all three modes, i.e., public, private and restricted mode. Our proposed DSMAM outperformed the performance in private mode in the best case, average case and worst case with respect to the existing IOTA MAM. In restricted mode, our proposed DSMAM performed similar to IOTA MAM while providing additional benefits of authenticity and security over the data using the dual signature. In public mode, the DSMAM performed poorer than the MAM in each of the categories, however it provided data authenticity. The comparison of latency between DSMAM and classical IOTA MAM version 0.x is shown in Table 8.

### 6.4. Challenges and Limitations

We have identified some technical limitations in our developed micro-payment over the top (MP-OTT) application as follows:

- Streaming video from cloud/database services to the client application was not within the scope of this project. This can be added as a feature for displaying multiple content on the OTT application. In our example, we had only one video by default in our application.
- No moving/play forward option was present in the video player at the proof of concept level. Since these have their own implementation complexity we will enhance our payment logic to tackle these situations in a later version.
- Problems of reuse of addresses exist in this system. Possible solutions are described below with insights into each solution.

**Figure 22:**
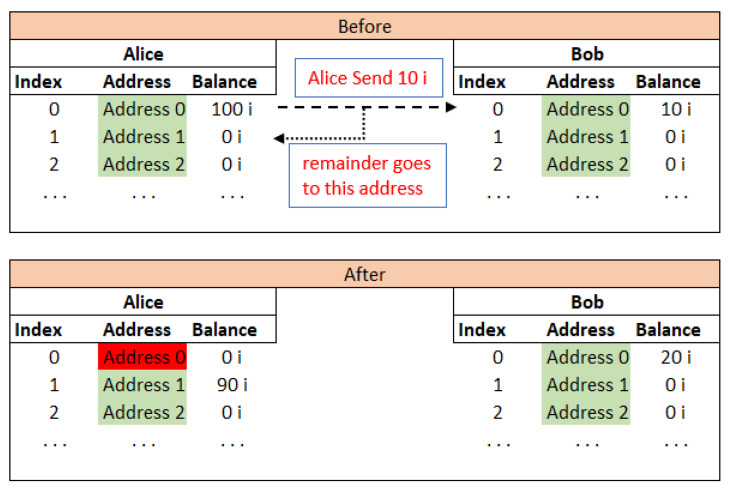
Problem of re-using used addresses.

Since the system uses W-OTS, it is must to use an address only once and not again [83]. This induces new challenges for the key and the address management task. For example, a donating address may be given by a non-government organisation (NGO), where any sender can donate iotas. But if the NGO ever uses this address for taking out the iotas out for any purpose, receiving future funds at this used address is not considered safe, so a new address known as remainder address has to be generated and shared with all the participants, which is not practical to implement. It might be easy to update the information between IoT devices about the new address but not possible in cases where people are in charge of those addresses. The problem is illustrated in Figure 22. Now, Alice should not use her Address 0 ever again. Bob can use his Address 0 any number of times for receiving funds, but once he uses this Address 0 to send any iotas, he cannot use his Address 0 again.

One possible solution to this is usage of reusable short address convenience (RSAC) as shown in Figure 7. This can act as an alias on behalf of IOTA addresses and will not change while an IOTA address at the backend can change, hence, making it useful in the transaction process several times even if addresses keep on changing. In contrast, no regular updates about the new address to other participants are needed. A summary of such a solution can be provided in the following way:

- Delion:api: (centralized-based solution): Maps E-mail-addresses as an alias with the master seed of the IOTA account holder (https://medium.com/delion-io/send-and-receive-iota-by-just-using-e-mail-addresses-9cf85bdb9bce, accessed on 12 March 2021).
- IOTA Cheque (proposed): uses a cheque book seed on top of current technology stack. Sender can send cheques to receivers. Fit for human-to-human interactions but not for an M2M economy (https://medium.com/iota-demystified/iota-cheques-sending-iotas-anywhere-without-requiring-a-receive-address-64570f42d6bc, accessed on 13 March 2021).
- IOTA Firefly wallet (presently active). Controlled by the IOTA Foundation as a fast payment method by saving data locally on user systems or phones and globally on the Tangle (https://firefly.iota.org/, accessed on 20 April 2021).
- Proposed Protocol RFC#0009 (proposed and under development in Coordicide Pollen (https://blog.iota.org/iota-2-0-introducing-pollen-nectar-and-honey-de7b9c4c8199/, accessed on 16 September 2020) Testnet [84] for IOTA 2.0) Based on using an Ed25519 signature as second signature scheme to prevent the transaction layout and address format in the present Tangle data structure with the current W-OTS (https://github.com/iotaledger/protocol-rfcs/blob/1d82efcd67895097ffabdb3f4fcb00f1646859f7/text/0009-ed25519-signature-scheme/0009-ed25519-signature-scheme.md, accessed on 13 December 2020).

### 6.5. Use and Application of the Proposed DSMAM and IBAVT

Decision-making behind the use of blockchain in any practical application can be realized through the Bart Suichies model (https://medium.com/block-chain/why-blockchain-must-die-in-2016-e992774c03b4, accessed on 25 April 2021). Alternatively, the IOTA platform aims to help the operational network engineers and blockchain architects in decision making to adopt IOTA if their use case and application require all the three aspects of ‘blockchain trilemma’ [85,86] which current blockchain platforms fail to provide.

However, in our opinion, the presence of a coordinator as already discussed in Section 3.3.3 in the IOTA network contradicts the decentralization parameter in the blockchain trilemma and acts as a limitation in the mass adoption of IOTA. Use cases and applications where IOTA can be adopted in the production use in the upcoming years are following:

- *Data Marketplaces*: ML Scientist or individuals depend on data from sensors and the IoT, especially in the case of the medical field. If data integrity is ensured then it boosts practitioners to work on the data. Further, any user can give access to whosoever he wishes to share data with.
- *Micropayments and Micro-finance Applications*: IOTA also has a built-in native cryptocurrency support named the same (iota) that can be used for micropayments in pay-as-you-go services.
- *Supply Chain and Logistics Applications*: Covid-19 has exposed several vulnerabilities of current supply chain systems where tracking and tracing for better transparency can be achieved by digitalizing the logistics to prevent another pandemic [87,88].
- *Industrial Internet of Things (IIoT)*: Real time applications are possible and data integrity and full access control is ensured.
- *Healthcare Applications*: People have control over their generated health-related data to prove data integrity and authenticity [89,90].
- *Data Centric Applications and Industry 4.0*: Any data centric application can use our enhanced architecture for securely storing data and for efficiently sharing it.
- *Decentralized Identity (DID) Protocols*: The IOTA platform can even further provide a trusted medium and protected environment for controlling decentralized identities for humans or things (https://www.iota.org/solutions/digital-identity, accessed on 30 April 2021) based on the (DIDs) specifications provided by W3C standards (https://www.w3.org/TR/did-core/#a-simple-example, accessed on 1 May 2021).
- *Decentralization and Access Control*: In this aspect nodes are not easily compromised and hacking is prevented by the use of PoW (Hashcash), which also prevents spam flooding of messages. Data integrity and access control are back in the hand of user.

A summary of these results is shown in Table 9.

## 7. Conclusions and Future Scope

Access control, data integrity and security are important considerations when developing IoT device applications. We fully implemented our proposed our two architecture viz. index-based address value transaction (IBAVT) and dual signature masked authenticated message (DSMAM) by referencing IOTA for the Internet of Things. We achieved robust, fast and efficient results as compared to the classical IOTA libraries for value-based and data-based transactions. IBAVT demonstrates the importance of address management in the IOTA platform to reduce the confirmation time. Our IoT sensor data visualizer app serves the need for visualization and further prepares data for processing using machine learning. The resultant system is modular in nature and easily interoperable with the current standard IoT implementations. We observed 24 instances of spam tests and presented the results. Throughout the study, we kept the IoT device constraints (low power IoT devices, packet bandwidth, etc) in mind and efficiently evolved the system for all shortcomings and challenges.

Reusable short address convenience (RSAC) is needed for dealing with the challenge of address reusability due to the use of the Winternitz One Time Signature scheme. Apart from that, this system can be helpful in healthcare, agriculture, defense, industrial IoT, commercial IoT, supply-chains, logistics, etc. for ensuring the veracity of the data and its authenticity while providing full access control ownership over data and devices. In our future work, we will explore new IOTA networks such as Chrysalis with other networks such as Lightning Network. We also aim to analyze the new IOTA Stream cryptographic framework that will offer high security for sending authenticated messages.

## Figures and Tables

**Table 1:** Summary and analysis of related research works on blockchain and IoT.

Ref. No.	Focus Area	Key Features	Remarks
[10]	Focus on the *data marketplace* for IoT data	Used smart contracts on Ethereum and through those users get access (swarm URL) to view data present on a decentralized storage SWARM.	Eliminates unreliable data providers. Uses a controlled environment for data sharing.
[11,12]	Focus on architectural frameworks like *Bitcoin but without mining and* *blockchain without PoW*	Presented a model of a block-based IoT architecture in which an overlay network is used along with cloud storage.	Several attacks and authentication properties are taken into consideration with respect to constrained IoT devices with a number of clusters in the network.
		Worked on removing proof of work in their proposed blockchain framework for IoT. They used Smart Home miner and an overlay network along with it.	They analyzed and evaluated the system based on energy consumption and time overhead.
[13]	Focus on *security and privacy* aspects of IoT devices	Presents a poposal for authentication and integrity for the Industrial Internet of Things (IIoT) that mitigate cyber-attacks using ECDSA signature algorithms between parties.	Based on DLT among several nodes, metrics are analyzed for different amount of node validators. Along with it, secure multi-party computation (SMPC) is used for grounded policy rules.
[14]	Focus on the *problem of synchronization* between IOT devices and policy controls	Used Ethereum Blockchain and three smart contracts for meter contracts for energy reading and another device is used as controller for air conditioners.	System is well synchronized as smart contracts are used but it is not fit for time-critical applications due to the long delays in transaction confirmation.
[15,16]	Provided *light blockchain node clients* for IoT devices.	Used Ethereum Blockchain using Solidity and Web3 JavaScript library for API’s to communicate	Extensive detailed architecture consisting of management hubs, managers, agent nodes, etc. Performance is tested on a constrained application protocol benchmark tool.
		The blockchain used is Ethereum and some modification is done to reduce the code size for light clients.	For performance and testing, the Wireshark benchmark tool is used to calculate the network bandwidth.
[17]	Focus on the *data marketplace* for IoT data	Used Ethereum as well as Monax along with a decentralized storage inter-planetary file system (IPFS).	Uses smart contracts on Ethereum and through that users get access (IPFS Image URL) to view data present on the IPFS. Presents a method for saving log files of blockchain in the full node that should be send to the IoT devices for synchronization
[18]	Did a *comparative study* between Ethereum and Hyperledger for IoT	Presented a theoretical model and practical comparison of DLT for IoT	Addressed key notions and techniques of different blockchain and DLT approaches for IoT with their uses and limitations.
[19]	Focus on *block performance evaluation*	They distinguished the network based on the size of blocks, number of IoT devices and the device location and then evaluation is performed.	Used a Bitcoin simulator to give metrics about the efficiency and throughout.
[20,21,22,23,24]	Focus on *scalability attaining high TPS*	They addressed scalability and reliability issues.	Presented the state-of-the-art of current blockchain for IoT solutions and the future trends.
		Used the public blockchain of Ripple Chain and ECA (based on keccak) as the signature algorithm	Settlement time observed was 3–5 s offering high TPS, although, it uses the same channel for both data and value and requires transaction fees which makes it unsuitable for IoT applications.

**Table 2:** Analysis of related research works on blockchain and M2M economy.

Ref. No.	Key Discussions
[60]	A blockchain-based P2P marketplace is created where the users can make transactions securely without any intermediary being involved.
[61]	Security issues in communications of CPS are highlighted and how blockchain technology can overcome such issues is discussed. To validate their claim a case study is also presented.
[62]	A novel charging and billing mechanism is proposed using the DLT- and IOTA-based micropayments. The proof-of-concept implementation was also provided.
[63]	A blockchain-based platform for the V2X economy named “Chorus” has been proposed which allows the entities of a V2X network to make transactions and other types of interactions in a P2P manner.
[64]	Provides a basic description and knowhow about IOTA Tangle. The description involves opportunities, issue and challenges in its implementation and widespread usage.
[65]	Discusses the importance of blockchain in reducing the trust tax. Specifically, in areas of supply chain for verifying the trustworthiness of steps involved and the authenticity of the final products, some kind of hidden cost is involved.
[66]	A protocol is discussed that reduces the transaction fees by aggregating multiple small value transactions into one larger transaction resulting into a single fee being applied. It measures the feasibility of using Bitcoin for an IoT (Raspberry Pi)-enabled machine to machine economy (smart cable and smart socket). They involve various setup modes such as standby mode, payed mode and lockout mode.
[67]	It provides an in-depth analysis of three aspects, namely benchmark performance comparison of multiple blockchain solutions with LN, integration of LN with the IoT ecosystem and it develops a novel payment algorithm designed for fee reduction. In their experimental period, LN out-performed sidechain and Bitcoin in IoT settings.
[68]	Their work focuses on three classifications of cryptocurrency solutions for machine to machine and consumer IoT. First classification is integration of IoT devices with major blockchain platforms such as Bitcoin and Ethereum where leverage of direct or light clients is discussed in comparison to full nodes. The second and third classification are a payment channel network (PCN) and newer IoT cryptocurrency proposals, respectively. PCN uses Lightning Network which is an off-chain transaction network solving scalability issues.
[69]	Lightning Network (LN) solves the issue of scalability but is not feasible to run it on IoT devices, hence the authors have demonstrated a 3-of-3 multisignature LN channel (i.e., the IoT device, the LN gateway and a bridge LN node) instead of a 2-of-2 LN channel. With their result, they showcased toll gate charge paymenta using their protocol for different vehicle speeds with a payment sending time of 2.55 s.
[70]	It solves the problem of transaction failures in the off-chain payments that could arise due to attacks or the absence of any node in the payment channel network between sender and receiver having multiple hops. Additionally, this paper also considers multi-path channels in the PCN to improve efficiency and robustness. If a payment is successful from one path leading to the receiver then the other paths will be invalidated.

**Table 3:** System and software specification.

Name of Software/Requirement	Purpose or Version
Node End-Point	Ubuntu 18.04, 6 Cores CPU, 16 GB RAM, 400 GB SSD (VPS)
Browser	Google Chrome
IDE	Visual Code Studio Editor
Language Used for Development	JavaScript
NodeJs and NPM	v12.16.2 and 6.14.4 respectively
IoT Devices used	Arduino Uno, ESP8266, Raspberry pi 3b
Sensor module used	DHT 11 (Digital Temperature Humidity) Sensor

**Table 4:** Latency achieved in IBAVT.

	Best Case	Average Case	Worst Case
Latency (in ms)	4931	5341	6097

**Table 5:** Result of the community spam test observed at our hornet node.

No. of Spamtest Instances	TPS	CTPS	Confirmation Rate
1	65.10	69	105.99
2	28	28	100.00
3	30.2	30.7	101.66
4	140	139	99.29
5	102	127	124.51
6	82	86	104.88
7	623.2	483.5	77.58
8	670	471	70.30
9	316	662	209.49
10	191	432	226.18
11	227	227	100.00
12	171	173.3	101.35
13	68	249.8	367.35
14	382.9	243.8	63.67
15	245.7	243.8	99.23
16	945.9	63	6.66
17	755	0	0.00
18	617	0	0.00
19	637	613	96.23
20	421	336	79.81
21	1019.4	912	89.46
22	907	813	89.64
23	143.6	147	102.37
24	141	136	96.45

**Table 6:** Performance comparison based on defined parameters.

Scheme	S	EC	CTPS	RO/C	SS	DI	AC	FE	TCC	DN	I/M
[10]	✓	✓	✘	✓	ECDSA	✓	✓	✘	✘	✓	✘
[12]	✓	✓	✓	✓	ECDSA	✓	✓	✓	✓	✓	✘
[13]	✘	✘	✓	✘	ECA	✓	✓	✘	✓	✓	✘
[14]	✘	✘	✘	✘	RSA	✓	✓	✘	✘	✓	✘
[15]	✘	✓	✘	✘	ECDSA	✓	✓	✘	✘	✓	✘
[17]	✓	✓	✘	✘	AES/DES	✓	✓	✘	✘	✓	✘
[24]	✓	✓	✓	✘	ECDSA	✓	✓	✘	✘	✓	✘
**Our Proposed Architecture DSMAM**	**✓**	**✓**	**✓**	**✓**	**EdDSA** **(ed25519)**	**✓**	**✓**	**✓**	**✓**	**✓**	**✓**

(S: Scalability; EC: Energy Consumption; CTPS: Confirmed Transaction per Second; RO/C: Reduced Overhead/Complexity; SS: Signature Scheme; DI: Data Integrity; AC: Access Control; FE: Fee-less Environment; TCC: Time Critical Computing; DN: Decentralized Nature; I/M: Interoperability/Modularity).

**Table 7:** Some notable Ed25519 features.

Feature	Remarks
Fast signing and verification	A quad-core 2.4 GHz Westmere (i3, i7, i9) signs 109,000 messages per second.
Fast Key Generation	/dev/urandom under Linux costs about 6000 cycles
High security level	Similar difficulty to break NIST P-256, RSA with 3000-bit keys, strong 128-bit block ciphers, etc
Collision resilience	Hash functions don’t let collisions break the system
No secret branch condition or caching	The operation of this is completely predictable. There is no chance of CPU caching or side channel attacks.
Small keys and signature	Public keys are usually 32 bytes and signatures are 64 bytes.

**Table 8:** Latency comparison between classical IOTA MAM and DSMAM.

	Classical IOTA MAM Latency (in ms)			DSMAM Latency (in ms)		
Mode	Best	Average	Worst	Best	Average	Worst
Public	3628	3716	5815	3741	3980	6604
Private	3662	3952	7232	3550	3875	6922
Restricted	3698	3682	7031	3645	3906	6851

**Table 9:** Performance output against research objectives.

Property	Output
CTPS/throughput	30–100 TPS (average case), 1100 TPS (best case)
Latency	5341 ms (5.3 s as average case) for value transactions and 3920 ms (3.9 s as average case) for data transactions. Best fit for time-critical IoT applications.
Network bandwidth	Up to VPS capability and at our VPS node, it was 400 Mbit/s
Size of network for scalability	Always high as it is directly proportional to more devices joining the network and increase in new tips.
Energy consumption for low power IoT devices	Low energy is required as remote PoW is enabled
Security	Free from man-in-the-middle attacks, and DDoS attacks. Feature like access control, security, data integrity, confidentiality all are preserved in the system.
Complexity (implementation)	Easy to develop, not as complex as Hyperledger Fabric-based IoT solutions

## Data Availability

Not applicable.

## Abbreviations

API	Application Programming Interface	MN	Master Node
ASCII	American Standard Code for Information Interchange	MWM	Minimum Weight Magnitude
CPS	Cyber Physical System	M2M	Machine to Machine
CTPS	Confirmed Transaction Per Second	NPM	Node Package Manager
DAG	Directed Acyclic Graph	OS	Operating System
DApp	Decentralized Application	OTT	Over The Top
DDoS	Distributed Denial of Service	PCN	Payment Channel Networks
DHT11	Digital Humidity Temperature 11 (Sensor)	PEM	Privacy Enhanced Mail
DLT	Distributed Ledger Technology	PoC	Proof of Concept
DSMAM	Dual Signature Masked Authenticated Message	PoW	Proof of Work
ECDSA	Elliptic Curve Digital Signature Algorithm	PoS	Proof of Stake
EdDSA	Edwards-curve Digital Signature Algorithm	P2P	Peer to Peer
HM-Kit	High Mobility Kit	RSA	Rivest Shamir Adleman
HN	Hornet Node	RSAC	Reusable Short Address Convenience
IBAVT	Index-based Address Value Transaction	SHA	Secure Hash Algorithm
IDE	Integrated Development Environment	SN	Slave Node
IoT	Internet of Things	SSH	Secure Shell
IPFS	Inter-Planetary File System	TPS	Transaction Per Second
IRI	IOTA Reference Implementation	URL	Uniform Resource Locator
LN	Lightning Network	URTS	Uniform Random Tip Selection
MAM	Masked Authenticated Message	VPS	Virtual Private Server
MCMC	Markov Chain Monte Carlo	V2X	Vehicle to Everything
ML	Machine Learning	W-OTS	Winternitz One Time Signature
MP-OTT	Micro-Payment Over the Top (Platform)	W3C	World Wide Web Consortium
